# ﻿Two new species of the *Clubionacorticalis* group (Araneae, Clubionidae) from Yunnan, China

**DOI:** 10.3897/zookeys.1224.135572

**Published:** 2025-01-28

**Authors:** Minghao Guo, Zhaoyi Li, Feng Zhang

**Affiliations:** 1 Hebei Vocational University of Industry and Technology, Shijiazhuang, Hebei 050091, China; 2 Key Laboratory of Zoological Systematics and Application, College of Life Sciences, Hebei University, Baoding, Hebei 071002, China; 3 Hebei Basic Science Center for Biotic Interaction, Hebei University, Baoding, Hebei 071002, China

**Keywords:** Clubionids, diversity, sac spiders, taxonomy

## Abstract

Two new species belonging to the *corticalis* group of the sac spider genus *Clubiona* Latreille, 1804 are described from both males and females: *Clubionalongyangensis***sp. nov.** and *Clubionamultiprocessa***sp. nov.** The two species are currently known to occur in Baoshan City and Dali Bai Autonomous Prefecture, Yunnan, China, respectively. Detailed descriptions, diagnoses, and photographs of the two species are provided.

## ﻿Introduction

The genus *Clubiona* Latreille, 1804 is widely known and distributed worldwide except for the polar regions (World Spider Catalog 2024). This genus comprises 79% of the total number of species of the family (528 of 665 described species) (WSC 2024). Due to the high species diversity of *Clubiona*, several infrageneric classifications have been proposed, and therefore *Clubiona* species have been assigned to a series of species groups and/or subgenera (e.g. Simon 1932; Dondale and Redner 1982; Deeleman-Reinhold 2001; Jäger and Dankittipakul 2010; Mikhailov 2012; Liu et al. 2016; Zhang et al. 2021; Wu et al. 2023).

There are at least 16 species groups of *Clubiona* discussed or frequently used in recent publications (Zhang et al. 2021). One of the most diverse groups is the *C.corticalis* group, which was first recognized by Simon (1932) and later by Mikhailov (1995), Deeleman-Reinhold (2001), and Yu and Li (2019). Until now, 86 *Clubiona* species have been assigned to the *C.corticalis* group, and they are mainly distributed in Eurasia and Australia (Zhang et al. 2021; Zhong et al. 2022; Li et al. 2023; Wu et al. 2023). Currently, 175 species of *Clubiona* have been recorded from China (WSC 2024), and 72 of them belong to the *corticalis* group (Zhang et al. 2021; Zhong et al. 2022; Li et al. 2023; Wu et al. 2023), making this group one of the most well-known clubionid groups in China.

While examining clubionid spiders collected from Yunnan Province, China, we encountered specimens of two undescribed species, *Clubionalongyangensis* sp. nov. and *Clubionamultiprocessa* sp. nov. These two species possess characters associated with the *corticalis*-group, but they can be easily distinguished from the other species in the group.

## ﻿Materials and methods

All measurements are given in millimeters. Leg measurements are shown as: total length (femur, patella, tibia, metatarsus, tarsus). Eye diameters as AME, ALE, PME, PLE and interdistances as AME–AME, AME–ALE, PME–PME, PME–PLE. Epigynes were removed and cleared in a pancreatin solution and then transferred to 75% ethanol for images captured. Photographs were taken using Leica M205A and Olympus BX51 microscope. All photographs were imported into Helicon Focus v. 7 for stacking. Final figures were retouched using Adobe Photoshop 2020. All specimens examined were deposited in Museum of Hebei University, Baoding, China (**MHBU**). The following abbreviations are used:

**A** atrium

**AER** anterior eye row

**ALE** anterior lateral eyes

**AME** anterior median eyes

**AME–ALE** distance between AME and ALE

**AME–AME** distance between AMEs

**B** bursa

**C** conductor

**CD** copulatory duct

**CO** copulatory opening

**E** embolus

**FD** fertilisation duct

**LTA** lateral tibial apophysis

**MOA** median ocular area

**PER** posterior eye row

**PLE** posterior lateral eyes

**PME** posterior median eyes

**PME–PLE** distance between PME and PLE

**PME–PME** distance between PMEs

**PPA** prolateral patellar apophysis

**RFA** retrolateral femoral apophysis

**RPA** retrolateral patellar apophysis

**RTA** retrolateral tibial apophysis

**S** spermatheca

**SA** spermathecal appendage

**SB** spermathecal base

**SH** spermathecal head

**VTA** ventral tibial apophysis

## ﻿Taxonomy

### ﻿ Clubiona corticalis

Taxon classificationAnimaliaAraneaeClubionidae

group

B9BE40AA-7B0E-58F3-9455-3984242060F6

Atalia Thorell, 1887: 54 (type species Ataliaconcinna Thorell, 1887).
Clubiona corticalis group: Simon 1932: 905; Mikhailov 1990: 142; Mikhailov 1995: 38, 42; Deeleman-Reinhold 2001: 90; Yu and Li 2019: 153.
Paraclubiona Lohmander, 1944: 19 (type species Araneacorticalis Walckenaer, 1802).

#### Diagnosis.

See Mikhailov (1995), Deeleman-Reinhold (2001), and Yu and Li (2019).

### ﻿ Clubiona longyangensis sp. nov.

Taxon classificationAnimaliaAraneaeClubionidae

361ABCDC-C5CF-5D3B-856F-AF0ABB5F7CDD

https://zoobank.org/E78D0880-A34C-404E-9D59-5B15FD1A75C6

**Figure 1.**
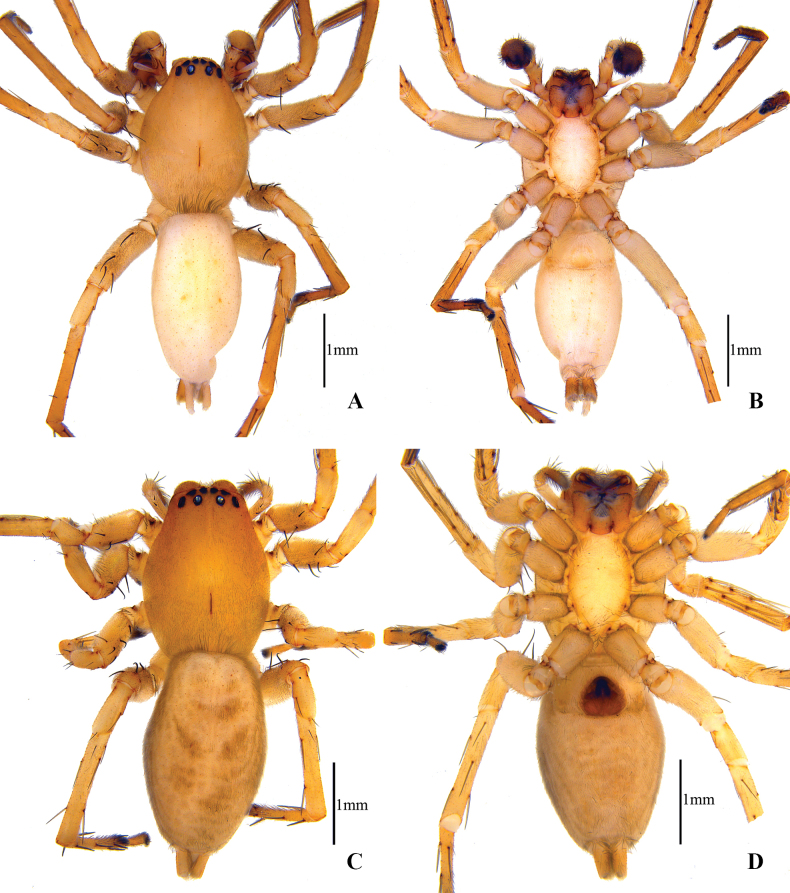
Habitus of *Clubionalongyangensis* sp. nov. **A** male (holotype), dorsal view **B** same, ventral view **C** female (paratype), dorsal view **D** same, ventral view.

**Figure 2.**
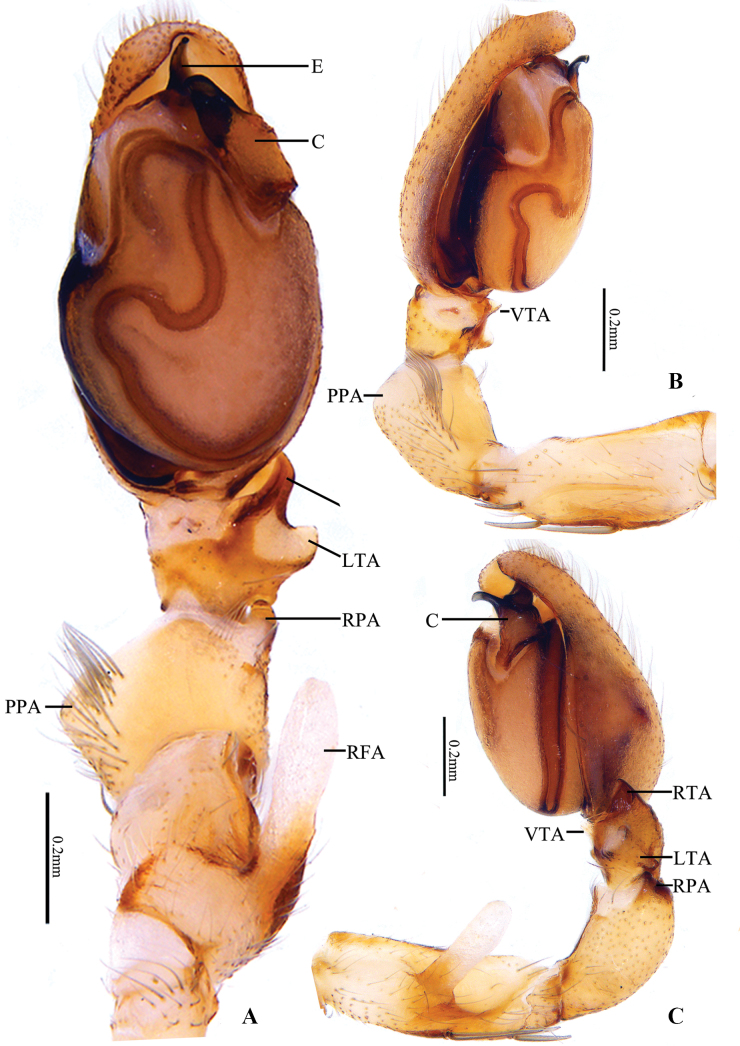
*Clubionalongyangensis* sp. nov., holotype male **A** left palp, ventral view **B** same, prolateral view **C** same, retrolateral view. Abbreviations: C = conductor; E = embolus; LTA = lateral tibial apophysis; PPA = prolateral patellar apophysis; RFA = retrolateral femoral apophysis; RPA = retrolateral patellar apophysis; RTA = retrolateral tibial apophysis; VTA = ventral tibial apophysis.

**Figure 3.**
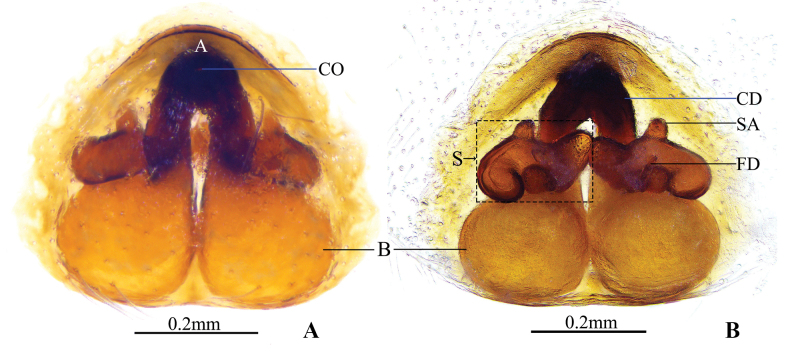
*Clubionalongyangensis* sp. nov., paratype female **A** epigyne, ventral view **B** vulva, dorsal view. Abbreviations: A = atrium; B = bursa; CD = copulatory duct; CO = copulatory opening; FD = fertilisation duct; S = spermatheca; SA = spermathecal appendage.

Figs 1 , 2 , 3 Chinese name: 隆阳管巢蛛

#### Type material.

***Holotype*** • ♂ (CLU847-1); China: Yunnan Province, Baoshan City, Longyang District, Lujiang Town, Baihua Ling Village; 25.3016°N, 98.7994°E; 1669 m elev.; 24.XI.2017; leg. Zhaoyi Li. ***Paratypes*** • 3♂2♀ (CLU847-2–CLU847-6); same data as holotype.

#### Other material examined.

3♂1♀ (CLU845-1–CLU845-4); Baihua Ling Village; 25.2981°N, 98.7863°E; 1983 m elev.; 24.XI.2017; leg. Zhaoyi Li.

#### Etymology.

The specific name is derived from the type locality; an adjective.

#### Diagnosis.

**Figure 4.**
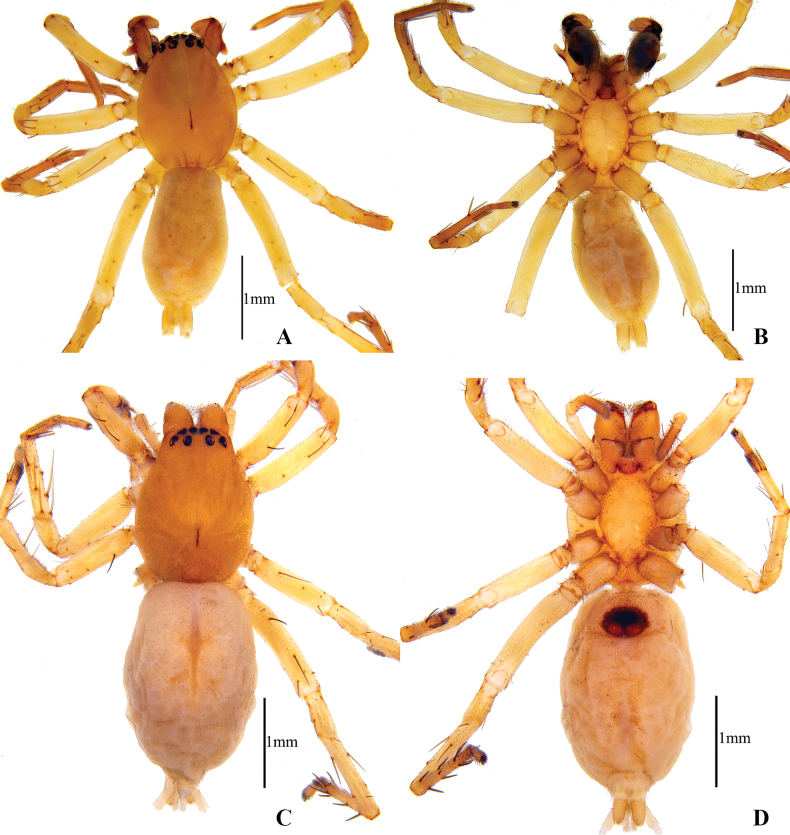
Habitus of *Clubionamultiprocessa* sp. nov. **A** male (holotype), dorsal view **B** same, ventral view **C** female (paratype), dorsal view **D** same, ventral view.

**Figure 5.**
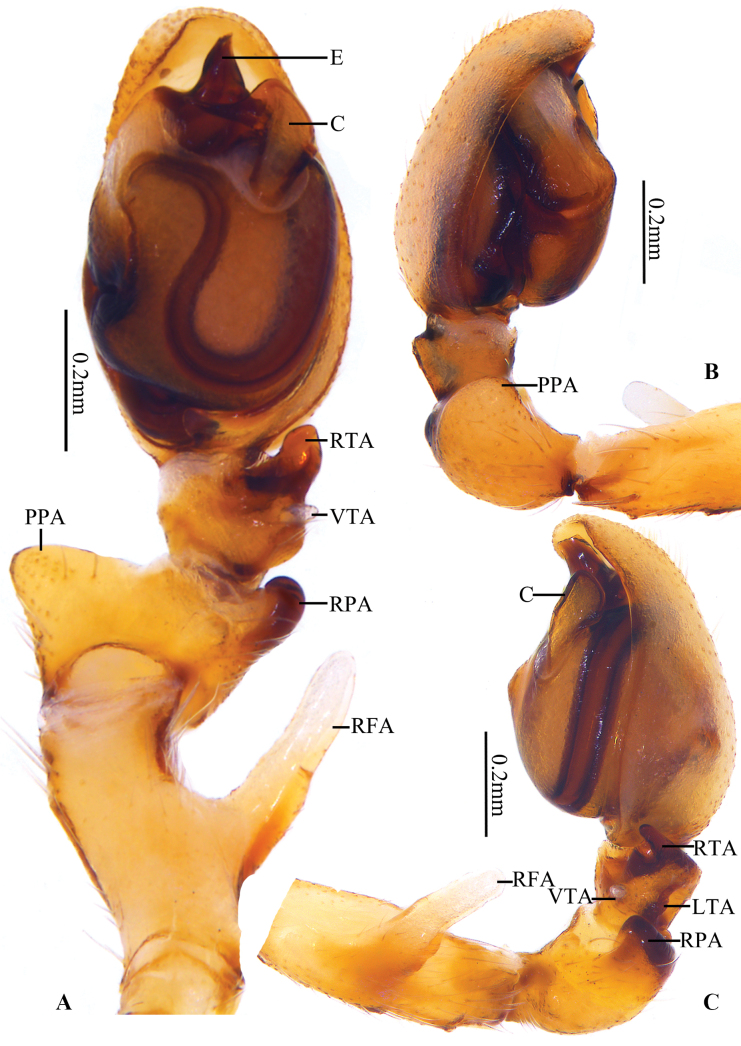
*Clubionamultiprocessa* sp. nov., holotype male **A** left palp, ventral view **B** same, prolateral view **C** same, retrolateral view. Abbreviations: C = conductor; E = embolus; LTA = lateral tibial apophysis; PPA = prolateral patellar apophysis; RFA = retrolateral femoral apophysis; RPA = retrolateral patellar apophysis; RTA = retrolateral tibial apophysis; VTA = ventral tibial apophysis.

Among the species of the *Clubionacorticalis* group, the male (Fig. 2) of this new species resembles *C.multiprocessa* sp. nov. (Fig. 5) by having a long (more than 1/2 of femur length), finger-shaped RFA (vs RFA absent or, if present, not finger-shaped in all other species in the grouper; e.g. tongue-shaped in *C.lamellaris* as shown by Zhang et al. 2018: figs 3C, 4C). However, the new species can be distinguished by the following: (1) VTA triangular, apex sharp (Fig. 2B, C) (vs papilliform; Fig. 5A, C); (2) LTA shaped like an inverted trapezoid, distal tip truncated in retrolateral view (Fig. 2C) (vs ridge-like; Fig. 5C); (3) conductor extends at a 45° angle towards the base of the embolus (Fig. 2A) (vs extends in a more or less 7-shaped; Fig. 5A); (4) embolus claw-shaped, with a curved apex in ventral view (Fig. 2A) (vs shaped like an equicrural triangle, embolic tip not curved; Fig. 5A). The female is similar to *C.falciforma* Liu, Peng & Yan, 2016 (Liu et al. 2016: 567, figs 30, 31, 35, 36) in the general shape of vulva (Fig. 3), but the new species can be distinguished by the following: (1) atrium umbelliform, with arched hood (vs elliptical and hood lacking); (2) bursae spherical (vs elongate-oval).

#### Description.

**Male (holotype)** (Fig. 1A, B): total length 4.50. Carapace 2.14 long, 1.52 wide; abdomen 2.36 long, 1.24 wide. Carapace yellowish brown, narrowed in pars cephalica, widest between coxae II and III, clothed with short fine hairs along the ridge of the thoracic region, forming a V-shaped region. Fovea longitudinal. AER slightly recurved, PER wider than AER, almost straight in dorsal view. Eye sizes and interdistances: AME 0.06, ALE 0.10, PME 0.08, PLE 0.10; AME–AME 0.05, AME–ALE 0.03, PME–PME 0.17, PME–PLE 0.11. MOA 0.28 long, front width 0.21, back width 0.36. Chelicerae reddish brown, with seven promarginal and five retromarginal teeth, with dense scopula in both margins. Clypeus height 0.05. Sternum light orange, 1.14 long, 0.71 wide. Labium and endites coloured as chelicerae, anterior edge with dark scopula, longer than wide. Legs yellowish brown, without distinct colour markings. Leg measurements: I 4.17 (1.13, 0.62, 1.35, 0.62, 0.45), II 4.24 (1.19, 0.63, 1.45, 0.52, 0.45), III 3.58 (1.01, 0.59, 1.02, 0.57, 0.39), IV 4.32 (1.25, 0.59, 1.49, 0.53, 0.46). Abdomen elongate-oval, dorsum pale yellow, with conspicuous anterior tufts of hairs, and two pairs of inconspicuous muscular depressions; venter pale yellow, with numerous yellowish spots.

***Palp*** (Fig. 2). Femur retrolaterally with a slanting finger-like apophysis (RFA), more than 1/2 of femur’s length. RFA partly membranous, arising mesially from femur, directing retrolatero-dorsally. Patella approximately twice as long as the tibia, with two apophyses: PPA large, broad, and blunt, located medially, represented by an enlarged tubercle; RPA short, almost thumb-shaped in ventral view, more or less inverted V-shaped in retrolateral view, located distally. Tibia short, with three apophyses: RTA short, with beak-shaped tip in ventral view, broad, flat, nearly triangular in retrolateral view, apex sclerotized; VTA short and membranous, almost triangular in lateral view; LTA shaped like an inverted trapezoid, distal tip truncated in retrolateral view. Cymbium almost 1.8 × longer than wide. Tegulum elongated oval, ca 1.6 × longer than wide; subtegulum visible prolaterally. Sperm duct long and sinuated, running an irregular course in the prolateral part of the tegulum. Embolus (E) originating at distal portion of tegulum, claw-shaped, apex curved ventrally. Conductor (C) large, originating from retrolateral side of tegulum, about 2 o’clock position, widest in the mid part, terminal part heavily sclerotized, beak-shaped, apex directing prolaterally.

**Female (paratype)** (Fig. 1C, D): one specimen body length 4.39. Carapace 2.10 long, 1.55 wide; abdomen 2.29 long, 1.53 wide. Carapace reddish brown, clothed with short fine hairs. Eye sizes and interdistances: AME 0.08, ALE 0.12, PME 0.09, PLE 0.10; AME–AME 0.06, AME–ALE 0.06, PME–PME 0.19, PME–PLE 0.15. MOA 0.30 long, front width 0.23, back width 0.39. Clypeus height 0.04. Sternum 1.24 long, 0.74 wide. Chelicerae, labium, and endites coloured as carapace. Leg measurements: I 3.76 (1.24, 0.41, 1.16, 0.56, 0.39), II 3.92 (1.20, 0.49, 1.12, 0.65, 0.46), III 3.47 (1.26, 0.47, 0.92, 0.56, 0.26), IV 4.15 (1.45, 0.58, 1.07, 0.68, 0.37). Abdomen oval, dorsum yellowish brown, with numerous, short fine hairs and two pairs of inconspicuous muscular depressions.

***Epigyne*** (Fig. 3). Epigynal plate slightly longer than wide. Atrium (A) distinctly large, umbelliform, with a delimited, arched anterior margin (hood) and nearly invisble postterior margin, located at anterior portion of epigynal plate. Copulatory openings (CO) indistinct, tiny, located centrally in atrium. Copulatory ducts (CD), heavily sclerotised, relatively long and thick, ca 1/2 length of epigynal plate, descend obliquely, forming a ˄-shaped course. Spermathecae (S) longer laterally juxtaposed at the tip represented by two ~-shaped tubes, anterior surface with a papilliform spermathecal appendage (SA), respectively. Fertilisation ducts (FD) acicular, located terminally on spermathecae. Bursae (B) close together, spherical, weakly sclerotized, surface smooth.

#### Distribution.

Presently known only from Yunnan, China.

### ﻿ Clubiona multiprocessa sp. nov.

Taxon classificationAnimaliaAraneaeClubionidae

9F72E0A2-9FDA-57BB-B05D-3F0F35EB9FF3

https://zoobank.org/86311755-A356-458D-8AA8-672B437D6514

**Figure 6.**
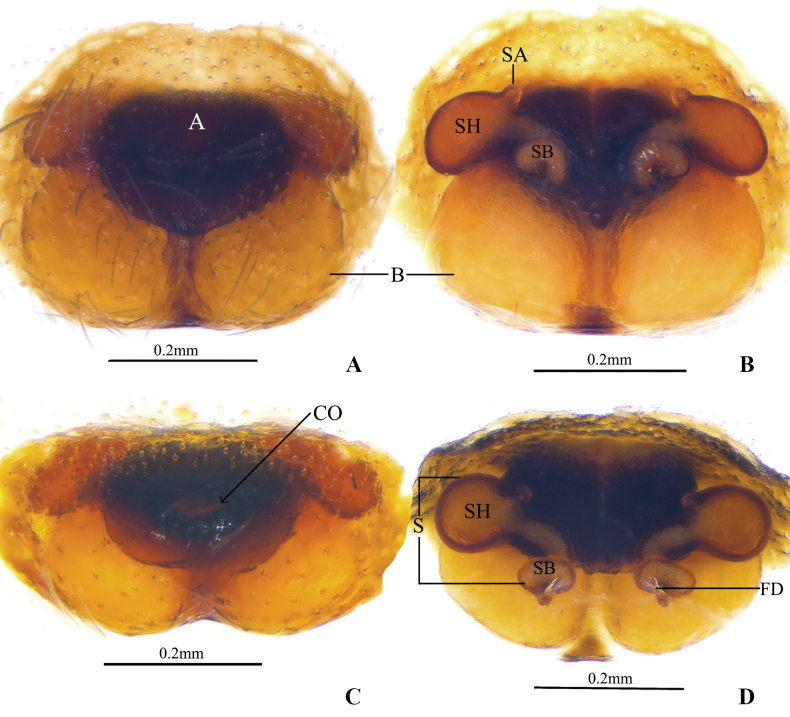
*Clubionamultiprocessa* sp. nov., paratype female **A** epigyne, ventral view **B** vulva, dorsal view **C** epigyne, anterior view **D** vulva, anterior view. Abbreviations: A = atrium; B = bursa; CO = copulatory opening; FD = fertilisation duct; S = spermatheca; SA = spermathecal appendage; SB = spermathecal base; SH = spermathecal head.

Figs 4 , 5 , 6 Chinese name: 多突管巢蛛

#### Type material.

***Holotype*** • ♂ (CLU1440-1), China: Yunnan Province, Dali Bai Autonomous Prefecture, Cang Shan; 2500 m elev.; 11.IX.2011; leg. Qiuju Wei. ***Paratypes*** • 2♂4♀ (CLU1440-2–CLU1440-7); same data as holotype.

#### Other material examined.

• 1♂9♀ (CLU1439-1–CLU1439-10), Cang Shan, 2500 m elev.; 11.VIII.2008; leg. Tingbang Yang • 1♂7♀ (CLU1441-1–CLU1441-8); Cang Shan; 2600 m elev.; 9.VIII.2011; leg. Qiuju Wei.

#### Etymology.

The specific name comes from the combination of “multi-” and “processus”, referring to the multiple (six) apophysis on the male palp; an adjective.

#### Diagnosis.

The male of *C.multiprocessa* sp. nov. can be distinguished from all other members of the *C.corticalis* group except for *C.longyangensis* sp. nov. (Fig. 2). Refer to the detailed diagnosis above for the similarities and differences between the two. The females of the new species (Fig. 6) resembles *C.applanata* Liu, Yan, Griswold & Ubick, 2007 (Liu et al. 2007: 64, figs 1, 2), *C.dichotoma* Wang, Chen & Zhang, 2018 (Wang et al. 2018: 319, figs 2C, D, 3F, G), *C.subapplanata* Wang, Chen, Zhang, 2018 (Wang et al. 2018: 327, figs 14C, D, 15E, F), and *C.lamellaris* Zhang, Yu, Zhong, 2018 (Zhang et al. 2018: 396, figs 3F, G, 4D, E) in the general shape of vulva, but the new species can be distinguished by the following: (1) epigynal plate wider than long (vs longer than wide in the other four species); (2) anterior surface of spermathecae with a papilliform appendage (vs absent in the other four species).

#### Description.

**Male (holotype)** (Fig. 4A, B): total length 3.34. Carapace 1.67 long, 1.21 wide; abdomen 1.67 long, 0.98 wide. Carapace uniformly orange-yellow, with indistinct radial striae. Fovea longitudinal, dark. AER slightly recurved, PER wider than AER, almost straight in dorsal view. Eye sizes and interdistances: AME 0.07, ALE 0.10, PME 0.08, PLE 0.11; AME–AME 0.03, AME–ALE 0.01, PME–PME 0.14, PME–PLE 0.10. MOA 0.26 long, front width 0.18, back width 0.31. Chelicerae yellow-brown, with four promarginal and five retromarginal teeth, with dense scopula in both margins. Clypeus height 0.02. Sternum pale yellow, 0.95 long, 0.63 wide. Labium coloured as chelicerae, anterior edge with dark scopula. Endites reddish brown. Legs pale yellow, tibia, metatarsus, and tarsus slightly darker in colour. Leg measurements: I 3.15 (0.85, 0.48, 0.72, 0.69, 0.41), II 3.69 (0.95, 0.45, 1.08, 0.78, 0.43), III 3.06 (1.07, 0.32, 0.64, 0.68, 0.35), IV 4.48 (1.46, 0.45, 0.84, 1.22, 0.51). Abdomen elongate-oval, dorsum yellowish brown, with conspicuous anterior tufts of hairs and many scattered darker spots.

***Palp*** (Fig. 5). Femur with a slanting finger-like retrolateral apophysis (RFA), ca 3/4 of femur’s length. RFA partly membranous, arising mesially from femur, directing retrolatero-dorsally. Patella with two apophyses: PPA broad and blunt, located medially, shaped like an equilateral triangle in ventral view; RPA short, ca 1/3 of patella’s length, slightly curved at apex in ventral view, more or less inverted V-shaped in retrolateral view, located distally. Tibia slightly shorter than patella, with three apophyses: RTA short and blunt, ca 1/2 of tibia’s length in ventral view, broad, flat, and with blunt apex in retrolateral view; VTA papilliform, short and transparent; LTA ridge-like, near the base of tibia. Cymbium almost 1.6 × longer than wide. Tegulum elongated oval, ca 1.4 × longer than wide; subtegulum visible prolaterally. Sperm duct sinuate, nearly U-shaped in ventral view. Embolus (E) arising from distal portion of tegulum, shaped like an isosceles triangle in ventral view, broad at base, gradually tapering toward apex. Conductor (C) large, originating from retrolateral side of tegulum, about 2 o’clock position, approximately 7-shaped in ventral view, distal part gradually tapering, extended transversely to the base of embolus.

**Female (paratype)** (Fig. 4C, D): one specimen body length 3.75. Carapace 1.68 long, 1.33 wide; abdomen 2.07 long, 1.47 wide. Carapace orange, clothed with short fine hairs. Eye sizes and interdistances: AME 0.08, ALE 0.09, PME 0.09, PLE 0.09; AME–AME 0.05, AME–ALE 0.04, PME–PME 0.18, PME–PLE 0.11. MOA 0.26 long, front width 0.21, back width 0.37. Clypeus height 0.02. Sternum 1.01 long, 0.64 wide. Chelicerae, labium, and endites coloured as carapace. Leg measurements: I 3.29 (0.96, 0.51, 0.88, 0.54, 0.40), II 3.45 (0.94, 0.53, 0.96, 0.63, 0.39), III 3.03 (0.74, 0.33, 0.85, 0.73, 0.38), IV 4.42 (1.29, 0.59, 1.14, 1.06, 0.34). Abdomen oval, dorsum greyish white, with a narrow longitudinal band in middle.

***Epigyne*** (Fig. 6). Epigynal plate slightly wider than long. Atrium (A) nearly heart-shaped, located at anterior portion of epigynal plate. Copulatory openings (CO) tiny, located centrally in atrium. Copulatory ducts (CD) invisible. Spermathecae (S) long, located at the anterior position of bursae, spermathecal heads (SH) ovate, with a papilliform spermathecal appendage (SA), spermathecal bases (SB) tubular, with small fertilisation ducts terminally (FD). Bursae (B) close together, nearly spherical, situated posteriorly.

#### Distribution.

Presently known only from Yunnan, China.

## Supplementary Material

XML Treatment for Clubiona corticalis

XML Treatment for Clubiona longyangensis

XML Treatment for Clubiona multiprocessa

## ﻿Additional information

### Author ORCIDs

Zhaoyi Li https://orcid.org/0000-0001-6303-6041

Feng Zhang https://orcid.org/0000-0002-3347-1031

### Data availability

All of the data that support the findings of this study are available in the main text.
